# The Role of Celebrities During the COVID-19 Pandemic in Iran: Opportunity or Threat?

**DOI:** 10.1017/dmp.2020.498

**Published:** 2020-12-22

**Authors:** Javad Yoosefi Lebni, Seyed Fahim Irandoost, Nafiul Mehedi, Sardar Sedighi, Arash Ziapour

**Affiliations:** 1 Community Medicine Department, School of Medicine, Lorestan University of Medical Sciences, Khorramabad, Iran; 2 Department of Public Health, School of Health, Urmia University of Medical Sciences, Urmia, Iran; 3 Department of Social Work, Shahjalal University of Science and Technology, Sylhet, Bangladesh; 4 Faculty of Physical Education, Sports Management Group, Tabriz University, Tabriz, Iran; 5 Health Education and Health Promotion, Health Institute, Kermanshah University of Medical Sciences, Kermanshah, Iran

**Keywords:** COVID-19, coronavirus, disease outbreaks

The 2019 coronavirus disease (COVID-19) spread rapidly from China to other countries around the world.^1,2^ Within a few months, it became the most pressing health problem in the world and was recognized by the World Health Organization as a pandemic.^2,3^ The death toll from this disease is rising every day, affecting all economic, social, and political dimensions of countries.^4,5^

On February 19, Iran joined the list of countries affected by the disease by recording the first cases.^6^ In total, according to the official statistics of the Ministry of Health of Iran, the number of people with COVID-19 in this country as of September 24 is 436 319, of which 25 015 people have died due to this disease and 367 829 people have been discharged from the hospital after a partial recovery.^7^

With the spread of COVID-19 in Iran and the beginning of quarantine, people were affected by financial and social problems, and various individuals and groups took actions to help. Celebrities are those who become popular in their social work in a field (such as actors, athletes, etc.). The media can play a vital role in introducing individuals and making them famous as celebrities.^8^ These days, celebrities are a kind of social authority for various social groups. Thus, their words, actions, and messages are effective. Celebrities can induce a part of society to follow and copy them, and their messages can influence the behavior of those people.^9^ Celebrities are one of the most important social groups that can play a significant role in resolving problems in various crises, due to their reputation and social authority, whether good or bad. In recent years, due to the growth of communication technologies, celebrities have been able to play a significant role in various crises, such as floods and earthquakes in Iran.^10,11^

Social media can provide valuable information quickly to large numbers of people about how to stay safe in a public health crisis like the COVID-19 pandemic. Yet social media, because it is totally free and its messages are not necessarily vetted by experts, can also spread false information and accusations, panic, and rumors, and thus make the crisis worse.^12^ As celebrities dominate social media, with their huge numbers of followers who regularly look for their messages, celebrities can make social media more of an asset or more of a problem in a crisis. Previous research has shown that celebrities can have a significant impact on people’s attitudes and beneficial behaviors in a crisis.^13,14^ In fact, the more popular a celebrity is, the more impact they can have on people’s behavior.^15^

There has been little research on the impact of celebrities on people’s behavior in the COVID-19 crisis in Iran or elsewhere. However, Mututwa and Matsilele showed that, although celebrities have sometimes published fake news about COVID-19 that have exacerbated the crisis, they can make a positive contribution by raising public awareness and concern.^16^

Unlike in other countries, where celebrities work formally and through the provision of resources to governmental and non-governmental organizations, in Iran, celebrities work individually and separately from official organizations and charities. It can create tension for them because, on the 1 hand, they are confronted with official institutions and are held accountable by them, and on the other hand, they may not be able to use the collected resources properly due to the lack of knowledge and awareness.

## Services and Activities Performed by Celebrities

### Positive Activities

#### Material Aid

This includes cash donations from their personal accounts, collecting donations from people and providing health supplies with these donations, donating a portion of their income and property to prevent and fight COVID-19, auctioning off their valuables and donating the money to poor families, and giving their properties to be used as hospitals or sanitary ware factories.

#### Personal Services

These services include the distribution of essential life-sustaining items, such as food, to people in need, categorizing and delivering aid to people, helping produce health products such as masks, hand sanitizers, making people laugh and entertaining them with their art such as dancing, creating a challenge (campaign) or competition on cyberspace, or showing their lifestyle to entertain people.

#### Fostering Awareness

Celebrities foster awareness through informing people about COVID-19, giving health advice on how to stay healthy, encouraging people to observe health precautions, and warning them about the dangers.

#### Supporting Medical Staff

Celebrities make appearances at hospitals, leaving comments and telling stories on the Internet to support medical personnel, and performing for hard-pressed medical staff.

### Negative Activities

#### Spreading Fake News

Among the false information spread by celebrities has included false reports of individuals’ deaths and about the general death toll in the country, false claims that other countries have a cure or treatment for COVID-19, and sensationalizing information to attract attention. With the outbreak of COVID-19, many celebrities’ shows and tours were shut down, giving them more time for social media. This has been a mixed blessing: more time to do good or to cause trouble.

#### Advertising of Drugs and Unhealthy Measures

Some celebrities have promoted quack cures and fake vaccines for COVID-19 on social media. Others have even encouraged violence. Some celebrities have advertised and even prescribed drugs to prevent COVID-19 that had not been proven safe or effective. Some celebrities sold medicines at excessive prices online, exploiting their fame and public confidence in them. Celebrities, lacking correct information, propagated the misuse of hygienic products. The misuse became widespread, and health service workers were diverted from patient care to deal with the effects and re-educated those who had been misled.

## Celebrities: Assets and Liabilities in the Pandemic Response

We can see that celebrities can play positive or negative roles in times of crises: sometimes both at the same time. Thus, celebrities are double-edged swords for those trying to manage a national crisis. Yet celebrities are a part of our social capital, and their social and personal capacities can be used to help in a crisis. Therefore, with more supervision over their activities and giving them correct information and guidance, celebrities’ social status can be used optimally for the benefit of society, and they can become more of an asset and less of a liability.
